# Protein Expression, Amplification, and Mutation of *HER2* Gene in Canine Primary Pulmonary Adenocarcinomas: Preliminary Results

**DOI:** 10.3390/ani14182625

**Published:** 2024-09-10

**Authors:** Barbara Brunetti, Dario de Biase, Francesca Millanta, Luisa Vera Muscatello, Enrico Di Oto, Roberta Marchetti, Ester Lidia Laddaga, Antonio De Leo, Giovanni Tallini, Barbara Bacci

**Affiliations:** 1 Department of Veterinary Medical Sciences, University of Bologna, 40126 Bologna, Italy; luisaver.muscatello2@unibo.it (L.V.M.); roberta.marchetti5@studio.unibo.it (R.M.); barbara.bacci@unibo.it (B.B.); 2 Department of Experimental, Diagnostics and Specialty Medicine, University of Bologna, 40126 Bologna, Italy; dario.debiase@unibo.it (D.d.B.); antonio.deleo@unibo.it (A.D.L.); giovanni.tallini@unibo.it (G.T.); 3 Department of Veterinary Sciences, University of Pisa, 56126 Pisa, Italy; francesca.millanta@unipi.it; 4 OaCP IE, Ltd., T12H1XY Cork, Ireland; enrico.dioto@live.it; 5 Ospedale Veterinario “I Portoni Rossi”, Anicura, Zola Predosa, 40069 Bologna, Italy; ester.laddaga@anicura.it; 6 Solid Tumor Molecular Pathology Laboratory, IRCCS Azienda Ospedaliero-Universitaria di Bologna, 40126 Bologna, Italy

**Keywords:** dogs, immunohistochemistry, fluorescence in situ hybridization, next-generation sequencing, lung, carcinoma, *HER2* gene, overexpression, amplification, mutation, V659S

## Abstract

**Simple Summary:**

Canine lung adenocarcinomas are malignant tumors generally treated by surgical excision. When inoperable, chemotherapy can be performed with limited benefit. For this reason, the use of targeted drugs can be attempted, as has already happened in human medicine. In this retrospective study, 19 canine lung adenocarcinomas were investigated with three different methods to evaluate the expression of HER2. This transmembrane receptor for the human epidermal growth factor is often altered in human epithelial tumors, for which many targeted drugs have recently been produced. Using immunohistochemistry, fluorescence in situ hybridization, and next-generation sequencing, we found 3 cases out of 19 with protein overexpression, 2 cases with amplification, and only 1 case with a specific type of mutation (V659E), probably sensitive to tyrosine kinase inhibitory drugs. Due to the similar HER2 molecular changes in dogs and humans, this study provides preliminary information regarding the possible future use of targeted therapies in canine pulmonary adenocarcinomas.

**Abstract:**

Recently, human epidermal growth factor receptor 2 (HER2) has emerged as a therapeutic target of interest for non-small-cell lung cancer in humans. The role of HER2 in canine pulmonary adenocarcinomas is poorly documented. To address this gap, this study employed three methodologies: immunohistochemistry (IHC), fluorescence in situ hybridization (FISH), and next-generation sequencing (NGS) to investigate the protein expression, gene amplification, and mutation of HER2 in 19 canine primary pulmonary adenocarcinomas. By IHC, 3 out of 19 cases were overexpressed 3+, 6 were 2+, and 10 were negative. With FISH, 2 cases were amplified (12.5%), 3 were inadequate for the analyses, and the others were non-amplified. With NGS, seven cases were inadequate. All other cases were wild-type, except for one IHC 3+ case, which was amplified with FISH and with a specific mutation already described in human pulmonary adenocarcinoma, V659E. This mutation is probably sensitive to tyrosine kinase inhibitory drugs. These results are similar to those in human medicine and to the few data in the literature on canine lung carcinomas; the presence of 12.5% of amplified cases in dogs lays the foundation for future targeted drugs against HER2 alterations.

## 1. Introduction

HER2 (also known as ERBB2) is a member of the human epidermal growth factor receptor (HER) family, a family of tyrosine kinase receptors that includes HER1 (EGFR or ERBB1), HER3, and HER4 [1]. In human medicine, HER2 is widely studied because mutations, overexpression, and amplifications have been reported in many types of cancer. For many of these (breast, gastric, bladder, and ovarian carcinomas), [2,3] HER2-targeted drugs are available, including trastuzumab, pertuzumab, lapatinib, and T-DM1 [2,3]. Lung cancer is the most common cause of cancer-related death worldwide. These tumors are divided into two major histological types: small-cell lung carcinoma (SCLS) and non-small-cell lung cancer (NSCLS), the latter comprising approximately 80–85% of all lung cancer cases [4]. In non-small-cell lung cancer (NSCLC), *HER2* overexpression and amplification have been described in 6–35% and 2–20% of patients, respectively [2,4]. Mutations of *HER2* are also reported in approximately 4% of patients [4,5,6]. Unfortunately, the first clinical trials with trastuzumab and other drugs failed to demonstrate benefits [2]. [4,5,6] In August 2022, based on data from DESTINY-Lung02, the FDA granted accelerated approval of the antibody–drug conjugate trastuzumab–deruxtecan, making it the first targeted agent approved for *HER2*-mutant NSCLC [6,7].

These conflicting results are partly due to the lack of patient selection and guidelines on evaluating HER2 alterations in NSCLC [6,7]. Only recently, in 2022, a consensus was published. The consensus established that NGS can be used for the *HER2* mutation test, FISH is recommended for the testing of *HER2* amplification, and IHC is recommended as the standard method for the detection of HER2 expression, considering positive cases of both 2+ and 3+ [7].

Primary lung tumors in dogs have a relatively low incidence. Around 2–4 dogs out of 10,000 develop primary pulmonary tumors in the US and UK, with most primary lung tumors being represented by carcinomas [8]. Canine lung adenocarcinomas are similar to human NSCLCs and include the same histotypes, such as adenocarcinoma and adenosquamous- and squamous-cell carcinoma [9]. In general, surgical removal by the lobectomy of relatively small solitary lesions has a good prognosis. In dogs with inoperable tumors, or in which surgery is otherwise contraindicated, chemotherapy with cisplatin and vindesine or doxorubicin or mitoxantrone has been used but with limited success [10]. In these latter cases, targeted therapies, as in human medicine, would be helpful. Only a few papers have investigated the molecular profile of these canine tumors, particularly regarding HER2 status [9,11,12].

Recently, Yoshimoto and coauthors described the expression of HER2 in 16 canine lung adenocarcinomas using immunohistochemistry. They found overexpression (3+) in 3 samples (19%) [12]. In 2019, Lorch and coauthors studied 88 primary canine lung tumors or cell lines using multiplatform next-generation sequencing. They found *HER2* point mutations in 38% of cases; the majority (93%) were hotspot V659E, transmembrane domain mutations comparable to activating mutations at the same site in human cancer. Other mutations were located in the extracellular domain and transmembrane. Moreover, they found that HER2^V659E^ cell lines displayed the constitutive phosphorylation of AKT and significantly higher sensitivity to the HER2 inhibitors lapatinib and neratinib [9].

This study aimed to analyze *HER2* amplification, mutation, and HER2 protein overexpression in 19 cases of primary pulmonary adenocarcinomas with the final goal of understanding the role of this oncogene in canine pulmonary adenocarcinomas. This is the first study to evaluate the amplification of *HER2* in canine primary pulmonary adenocarcinomas using FISH analysis. It also examined the expression of HER2 using three different methodologies in the same cases.

## 2. Materials and Methods

Primary pulmonary adenocarcinomas from 19 dogs from the Anatomic Pathology Service of the Department of Veterinary Medical Sciences, the University of Bologna, and the private histopathology laboratory of the AniCura Veterinary Hospital “I Portoni Rossi” in Zola Predosa (Bologna, Italy) were retrospectively collected. 

The experiments complied with the national legal treatment of animal tissue samples. The samples were surgical specimens available as formalin-fixed paraffin-embedded (FFPE) material, routinely stained with hematoxylin and eosin (H&E). These specimens were sent from internal hospitals or external private clinics for diagnostic purposes. The samples were received as formalin-fixed samples; the fixation times were unknown.

The slides were reviewed by two board-certified authors (BBr and BBa) until a consensus diagnosis was reached, and the histological subtype and grading were assessed according to the classifications of Wilson et al. (2016) [10], McNiel et al. (1997) [13], and Caswell and Williams (2016) [14]. We selected cases of primary canine adenocarcinomas from 2016 to 2023, excluding cases in which only biopsies were available. Biopsies were not used because they are often in such quantities that it is not possible to use the three different methods to detect HER2 alterations.

### 2.1. Immunohistochemistry

The antibody for HER2 (polyclonal A0485, Dako, Glostrup, Denmark; dilution 1:200) was already validated for cross-reactivity in dogs [15,16]. The immunohistochemical method was performed as previously published [16,17]. Sections of canine mammary carcinomas previously scored as 3+ for HER2 were used as positive controls to assess the specificity of the immunohistochemical procedure. Moreover, as already reported [9], the normal bronchiolar and bronchial epithelia were used as additional positive internal controls. The primary antibody was replaced with an irrelevant, isotype-matched antibody in the negative control.

The evaluation of the immunohistochemical expression of HER2 was recorded according to the current recommendations of the American Society of Clinical Oncology/College of American Pathologists (ASCO/CAP) [18]. The ASCO/CAP algorithm is based on the cellular membranous intensity of staining and the percentage of positive tumor cells (with a cut-off value of 10%). Immunoreactivity to HER2 was divided into the following classes: 3+ (positive), 2+ (equivocal), and 0 and 1+ (negative). HER2 immunoreactivity was also evaluated following the consensus for HER2 alterations in NSCLC [7], considering 0 and 1+ as negative and 2+ and 3+ as HER2-positive cases.

In the statistical analysis, we tried the analysis considering both IHC 2+ and 3+ as positive (P) and 0 and 1+ as negative (N).

### 2.2. Fluorescence In Situ Hybridization Method

We used tissue microarrays (TMAs) for the FISH analyses, built according to the previously published method [16,17,19]. Six double-core TMA blocks were built. Each TMA tumor block included 6 or 7 cases. The cores were asymmetrically arranged, and a different tissue (liver) was included as a negative control and orientation core.

OaCP IE LTD (Phoenix House, Monahan Road, T12H1XY, Cork, Ireland) provided specific probes for the HER2 gene’s identification and properly mixed them with the in situ hybridization buffer SMART-ISH Solve (OaCP IE Ltd., Phoenix House, Monahan Road, T12H1XY, Cork, Ireland).

The FISH method was performed based on a previously published method [16]. Canine mammary carcinoma with HER2 amplification was used as a positive control. The specificity of FISH was evaluated considering the euploidy of the fibroblasts and lymphocytes near the neoplastic cells. The image analysis software was cytogenetic specific (CytoVision^®^, Leica Biosystem, Nussloch, Germany) and was used to count the number of gene copies per nucleus in at least 20 tumor nuclei for each tumor, choosing the most representative area. Fluorescent hybridization signals were visualized using a fluorescence microscope (Olympus BX6T, Hachioji, Tokyo, Japan) equipped with ×100 oil immersion objectives and appropriate filters (spectrum orange for locus-specific probe HER2, spectrum green for locus-specific probe CRYBA1, and UV filters for the DAPI nuclear counterstain). The evaluation system used to interpret FISH sections was based on the ASCO/CAP guidelines (2018) [20] using a double probe, as described in our previous paper [16].

### 2.3. Next-Generation Sequencing (NGS)

All cases were analyzed with NGS. NGS was performed at the Molecular Pathology Laboratory of Solid Tumor of the Policlinico Sant’Orsola-Malpighi, Bologna, Italy. Only tumor areas with a more significant tumor cellular component were selected, and the DNA was extracted from two to three 10 um thick tissue sections using the Quick Extract FFPE Kit (Lucigen, LGC Biosearch Technologies, Hoddesdon, UK). According to the manufacturer’s recommendations, the DNA was quantified using a Qubit fluorometer (Thermo Fisher Scientific, Waltham, MA, USA). The DNA was amplified using a laboratory-developed NGS panel (Molecular Pathology Laboratory of the Policlinico Sant’Orsola-Malpighi), as previously described [21]. The custom panel was designed using the Ion AmpliSeq Designer Tool (Thermo Fisher Scientific) covering the coding sequences of the following genomic regions (reference: Canis lupus familiaris 3; a total of 92 amplicons between 125 and 175 bp in length; panel size: 9.33 kb): *HER2* (start chr9: 22785066; end chr9: 22759630) and *CRYBA1* (start chr9: 43372505; end chr9: 43378500).

The sequencing was performed using the GeneStudio S5 Sequencer (Ion 530 Chip), and the results were analyzed using the Ion Reporter plugin Variant Caller (Thermo Fisher Scientific) and the Integrated Genome Viewer software (IGV v2.12.2, http://software.broadinstitute.org/software/igv/) (URL accessed on 23 November 2023). To avoid false-positive results, only the nucleotide variants with a variant allele frequency (VAF) higher than 10% were used for the mutational call [21]. The functional effect of each variant was evaluated using the PolyPhen2 tool (http://genetics.bwh.harvard.edu/pph2/) (URL accessed on 23 November 2023).

### 2.4. Follow-Up Data

Lymph node metastasis was detected by histological examination when the lymph node was available. Survival status was investigated and collected by phone calls to the referring veterinarian or the dog’s owner. Patients were followed up after study inclusion for 1 year and were recorded as dead or alive. 

### 2.5. Statistical Analysis

Statistical analysis was performed with R version 4.2.0. The mean and standard deviation (SD) were calculated for normally distributed data, while the median (min–max) was reported for non-normally distributed data. Correlations were performed with chi-square tests for categorical variables. Values of <0.05 were considered statistically significant.

## 3. Results

This study was conducted on a retrospective caseload, selecting 19 dogs with primary pulmonary adenocarcinomas. The patients were three intact females, six spayed females, two intact males, and eight neutered males. The mean age of the patients at diagnosis was 11.6 years ± 2.32 (mean and standard deviation). Nine dogs were mixed-breed: two Labrador Retrievers, two Fox Terriers, and one Bull Terrier, Weimaraner, Bloodhound, Rottweiler, Shih-Tzu, and Chihuahua.

**Figure 1:**
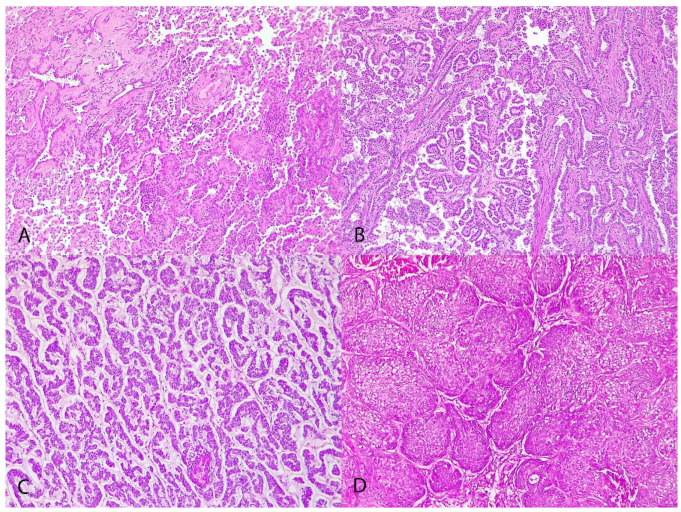
Canine pulmonary adenocarcinomas. (**A**) Lepidic pattern; H&E; objective 10×; dog n 15. (**B**) Papillary pattern; H&E; objective 10×; dog n 19. (**C**) Micropapillary pattern; H&E; objective 10×; dog n 12. (**D**) Squamous pattern; H&E; objective 10×; dog n 17.

Histologically, tumors were classified as lepidic (2/19, 10.52%) (Figure 1A), papillary (13/19, 68.42%) (Figure 1B), micropapillary (1/19, 5.26%) (Figure 1C), and squamous (3/19, 15.78%) (Figure 1D). Tumors were grade I in 7 cases (36.8%), grade II in 10 (52.6%), and grade III in 2 cases (10.5%).

In 9 cases, the examined lymph nodes were negative for metastasis; in 2 cases, there were metastases (2/11, 18.18%); and in the other cases, the lymph node was not examined. Follow-up data were available in 17/19 cases. All the results are presented in Table 1. There was no statistically significant association between the histological classification and histological grading (Fisher’s test; *p* = 0.259). The histological classification and grading were not significantly correlated with 1-year survival (*p* = 1 and *p* = 0.38, respectively; Fisher’s test).

### 3.1. Immunohistochemistry

Normal bronchiolar and bronchial epithelia have moderate cytoplasmic or membranous labeling for HER2. The positive control, a canine mammary carcinoma with an IHC 3+ score, showed intense and membranous positivity in more than 10% of neoplastic epithelial cells.

**Figure 2:**
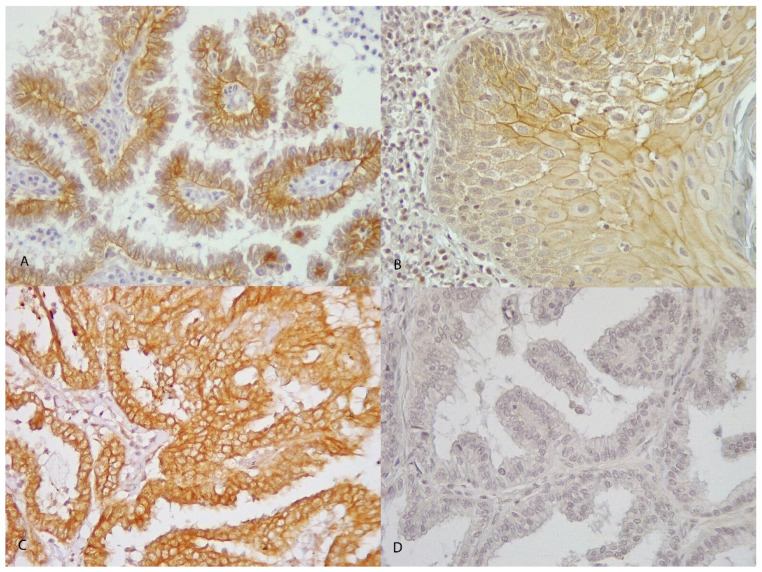
Immunohistochemistry for HER2 in canine pulmonary adenocarcinoma. (**A**) There is an incomplete membrane staining of tumor cells in more than 10% of tumor cells; HER2 score of 1+; papillary carcinoma; objective 40×; dog n 13. (**B**) Weak–moderate complete and circumferential membrane staining in more than 10% of tumor cells; HER2 score of 2+; squamous carcinoma; objective 40×; dog n 17. (**C**) Intense circumferential membrane staining in more than 10% of tumor cells; HER2 score of 3+; papillary carcinoma; objective 40×; dog n 19. (**D**) No staining is observed; HER2 score of 0; papillary carcinoma; objective 40×; dog n 1.

A total of 3 out of 19 carcinomas (15.8%) showed a complete intense membrane labeling for HER2 in >10% of tumor cells and were considered positive (3+ score) (Figure 2C). A total of 6 out of 19 carcinomas (31.6%) were interpreted as equivocal (2+ score), showing complete moderate or scant HER2 expression (Figure 2B). Negative cases accounted for 10 out of 19 (52.63%), either showing weak incomplete membrane expression (1+ score; 4/19 (21.1%)) (Figure 2A) or no immunoreactivity (0 score; 6/19 (31.6%)) (Figure 2D). There was no correlation between HER2 IHC 3+ and the histological classification (*p* = 0.70); neither considered 2+ and 3+ as positive and 0 and 1+ as negative (*p* = 0.58). There was no correlation between HER2 IHC 3+ and the histological grade (*p* = 0.36); neither considered 2+ and 3+ as positive and 0 and 1+ as negative (*p* = 0.65). HER2 IHC 3+ expression was not associated with 1-year survival (*p* = 0.77; Fisher’s test), neither considering 2+ and 3+ as positive and 0 and 1+ as negative (*p* = 0.57; Fisher’s test).

### 3.2. Fluorescence In Situ Hybridization

A total of 3 out of 19 pulmonary carcinomas that underwent FISH analysis were technically inadequate (non-evaluable) and, therefore, considered underdetermined (1 was a 3+ with IHC).

**Figure 3:**
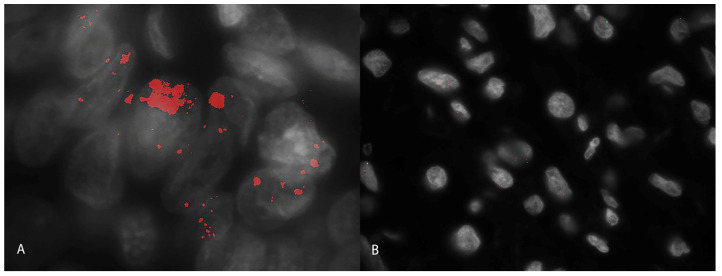
Canine pulmonary adenocarcinomas; lung; dog. (**A**) High magnification of nuclei with HER2 amplification (cluster-type amplification) in a papillary adenocarcinoma with FISH analysis; dog n 3; many copies of *HER2* (red signal) are visible in the neoplastic nuclei. Red: *HER2* gene. Green: *CRIBA1* gene. White: DAPI. (**B**) High magnification of nuclei with diploid hybridization signals for the *HER2* gene and *CRIBA1* gene with FISH analysis; dog n 16. Red: *HER2* gene. Green: *CRIBA1* gene. White: DAPI.

We used a dual-probe evaluation and considering the combined signals of *HER2* (a red signal) and *CRYBA1* (a green signal), 2 (2/16; 12.5%) carcinomas were amplified (Figure 3A), and 14 (14/16; 87.5%) carcinomas were not amplified (Figure 3B). The two amplified cases were papillary carcinomas, and both showed a cluster-type amplification. Cluster-type amplification was characterized by numerous, closely stippled adjacent signals forming a large visible cluster, and it was interpreted as amplification of the *HER2* genes with a conventional ratio with *CRIBA1* of more than 10.

No significant association was found between the *HER2* amplification status and histological classification (Fisher’s test; *p* = 1), nor with the histological grading (Fisher’s test; *p* = 1). The *HER2* gene amplification and HER2 IHC 3+ expression were not significantly correlated (Fisher’s test; *p* = 0.458), neither considering 2+ and 3+ as positive and 0 and 1+ as negative (*p* = 0.175). No significant correlation was observed between FISH and 1-year survival (*p* = 0.28; Fisher’s test).

### 3.3. Next-Generation Sequencing (NGS)

For the present scientific work, only the HER2 panel was considered. The panel was designed to sequence the entire gene’s CDS (coding sequence). Therefore, it was expected to detect almost all possible variants in the HER2 gene.

For the sequencing, 7 out of 19 (36.8%) were found to be non-evaluable due to high DNA degradation; unfortunately, among these, there was an equivocal case of 2+ in IHC and amplified with FISH. A total of 11 out of 12 (91.66%) were wild-type. Among these, two cases were found to be 3+ in IHC. Only 1 case was mutated (1/12; 8.3%) with a particular kind of mutation indicated as V659E (variant allele frequency (VAF): 29%); this case was found to be 3+ by IHC. This V659E variant was predicted as “probably damaging” (score: 1.000) using the PolyPhen 2 tool. Only one case was not evaluable with FISH and simultaneously with NGS; the other cases that were not evaluable either had FISH or NGS. All results are reported in Table 1. Statistical analysis was not performed due to an insufficient number of mutated cases.

## 4. Discussion

Primary lung tumors in dogs have a relatively low incidence within the pet population; therefore, the numerosity of the studies is limited. In this investigation, we analyzed the HER2 status with three different methods in 19 primary pulmonary adenocarcinomas in dogs. In the literature, only two studies have analyzed HER2 expression in canine pulmonary adenocarcinomas. The paper published by Yoshimoto and coauthors evaluated the expression of HER2 with IHC in 16 canine pulmonary carcinomas, finding 3 samples (19%) scored 3+ (overexpressed), 8 cases (50%) 2+ (equivocal), and 5 (31%) cases 1+. The authors applied the ASCO/CAP (2013) evaluation but included the equivocal (2+) cases as positive, anticipating the indications provided by the 2022 consensus [7,12]. Moreover, the authors evaluated the mRNA expression in six fresh canine pulmonary carcinomas and four normal lungs, finding that five out of six carcinomas had significantly higher HER2 mRNA expression than in normal samples. Our immunohistochemistry results are similar as we also had 3 cases considered 3+ (3/19; 15.7%) and 6 cases 2+ (6/19; 31.57%), and the remaining cases were negative. If we consider the results obtained in human medicine in lung carcinomas in which the percentage of 3+ IHC cases for HER2 varies from 6 to 35%, our study and that of Yoshimoto and coauthors present comparable values [12,22].

Compared with our work, Yoshimoto and coauthors [12] used a different antibody (monoclonal antibodies, clone, CB-11, Leica Biosystems, Wetzlar, Germany), and they also considered the equivocal cases (2+) positive, as already reported in the consensus and some older studies [7,23,24]. In our study, we performed statistical analysis excluding and including 2+ as positive cases, but we did not find any significant correlation with other parameters. Since only two publications have analyzed immunohistochemical HER2 expression in canine lung tumors, it is uncertain whether to consider only the 3+ subgroups positive. More studies will be necessary to make this decision.

In human medicine, it is controversial to apply the ASCO/CAP guidelines for mammary carcinomas to lung carcinoma and, in particular, to NSCLCs [7]. In 2022, a consensus for HER2 testing in human NSCLCs was published [7]. They stated that IHC is recommended as a standard method for the detection of HER2 expression in solid tumors such as breast cancer, gastric cancer, intestinal cancer, and NSCLC. However, HER2 expression is not routinely tested in clinical practice for NSCLC. Intriguingly, there is no apparent correlation between *HER2* amplification and overexpression in NSCLC, which is in sharp contrast with what is observed in breast cancer. The same consensus states “FISH confirmation is not required for NSCLC patients with HER2 IHC 2+/3+ to define positive HER2 expression” [7]. Studies demonstrate that HER2 protein expression in NSCLC is different from breast cancer: In a study designed to elucidate the concordance between HER2 IHC and in situ hybridization in NSCLC, the results showed that the concordance rate of the HER2 IHC (2+) subgroup was 0.091, which was much lower than 0.975 found in the HER2 IHC (0/1+) subgroup and 0.665 for the HER2 IHC (3+) subgroup [23]. In another study, the sensitivity and specificity of IHC for detecting amplification were 23.9% and 94.9% at a cut-off of more than or equal to 2+, respectively [25].

Lorch and coauthors analyzed HER2 in 88 primary canine lung tumors or cell lines with NGS, HER2 RNA, and protein expression by qRT-PCR and IHC [9]. RNA samples from 49 lung tumors were evaluated alongside 14 normal lung tissue samples distal to tumor areas but from the same lung lobe. For HER2 RNA expression, no significant difference was found in the relative HER2 expression between the tumor and normal or HER2-mutant and HER2^WT^ groups. Only eight lung carcinomas were examined with IHC using the Visiopharm Image Analysis software (Visiopharm, Hørsholm, Denmark version 2017.27.0.3313), finding that all cases (eight) were positive for HER2, with homogeneous and diffuse labeling of the tumor cell cytoplasm and cell membrane [9]. Moreover, with NGS analysis, they found *HER2* point mutations in 38% of canine lung adenocarcinomas, and 93% of these *HER2* mutations were hotspot V659E transmembrane domain (TMD) mutations, comparable to activating mutations at this same site in human cancer. They also found that *HER2^V659E^* cPAC cell lines displayed constitutive phosphorylation of AKT and significantly higher sensitivity to the HER2 inhibitors lapatinib and neratinib relative to *HER2*-wild-type cell lines [9]. We found the same type of mutation with NGS analysis in only one case, the IHC 3+ (overexpressed) case, concurrently amplified with FISH. These results contradict those found in human lung adenocarcinomas, where *HER2* amplifications were not associated with *HER2* mutations [2]. Conversely, we found a mutation and amplification of the *HER2* gene in the same case. In our series, only 1 case out of 12 was mutated (8.3%), a lower percentage than that found in the paper by Lorch and coauthors [9] but higher than those found in human medicine [5].

This is the first work to analyze *HER2* amplification in canine pulmonary adenocarcinomas. We found, with FISH analysis, that 2 cases out of 16 (12.5%) were amplified, similar to the percentages found in human lung carcinomas (2–20%) [22]. Moreover, these two cases had a specific cluster-type amplification, which indicated many amplifications of the *HER2* gene. In our caseload, 3 out of 19 cases were 3+ with IHC, and 6 were 2+ with IHC. Of these, only one 3+ case and only one 2+ case were amplified.

A weak point of this work was the presence of cases unsuitable for FISH (three cases) and NGS analysis (seven cases). Unfortunately, a FISH-amplified and IHC2+ case was not evaluable with NGS. The difficulties related to FISH analysis can be due to pre-analytical factors. In particular, over-fixation in formalin can reduce the hybridization efficiency. Moreover, nuclear digestion can influence the final results [26]. The same considerations are valid for NGS analysis, in which the main issue is the inability to extract sufficient DNA from the FFPE material. This may be due to pre-analytical conditions, such as prolonged formalin fixation [21,27,28]. Unfortunately, the problem of over-fixation in veterinary medicine is complex; many samples come from single private practices and are not always sent immediately to the histopathology laboratory. In human medicine, everything is standardized because therapies are based on the results of IHC, FISH, and NGS, while in dogs, for these studies, we are still at the experimental research level. Therefore, the method most affected by DNA degradation was NGS, with seven non-evaluable cases. By combining the NGS results with FISH analyses, almost all cases were evaluated for HER2 mutation/amplification, and only one case remained unevaluable.

As shown in Table 1, the three cases found to be 3+ overexpressed were as follows: one amplified and mutated, one non-amplified and wild-type, and one wild-type. In these last two cases, the cause of IHC overexpression must have differed from amplification, or a different mutation may have increased the stability of the protein. It is also possible that post-transcriptional modifications or either up-regulation or down-regulation of miRNAs affect the expression of the HER2 protein [29].

Although the case series of this study was limited, and some cases could not be evaluated with FISH and NGS, some considerations regarding the methods can be made. Each technique provides valuable information to understand the role of HER2 in cells. With immunohistochemistry, we can identify the cases that could benefit from therapies, mainly only 2+ and 3+ cases. None of the negative IHC cases had amplifications or mutations. With FISH, indeed, we can identify amplified cases, and with NGS, if particular types of mutations are present. This is the first study to demonstrate the presence of *HER2* amplification in canine lung adenocarcinomas with FISH and also demonstrates that amplification may be associated with a particular type of mutation, the *HER2^V659E^* mutation, which can potentially benefit from targeted therapy.

Further studies with larger case series will be necessary to better understand the role of HER2 in canine pulmonary adenocarcinomas and the potential use of HER2 inhibitors in dogs.

## 5. Conclusions

In conclusion, the papillary pattern was the most frequent histotype in canine lung adenocarcinomas. Most cases were grade II. Considering the HER2 analysis, a minority of the cases were IHC-overexpressing, and excluding the non-evaluable cases, half of these were amplified. Cases that are 2+ require further investigation, and FISH must be performed to check whether there is amplification. Only one in five (2+) was amplified in our series.

Moreover, *HER2^V659E^* mutation was found with NGS in an IHC 3+ and amplified case, suggesting that in canine pulmonary carcinoma, *HER2* mutation and amplification may not be mutually exclusive and that these cases may benefit from therapy with HER inhibitors.

## Figures and Tables

**Table 1:** Clinical, histological, and molecular characteristics of 19 canine primary pulmonary carcinomas.

n	Breed	Sex	Age	Histological Classification	Histological Grading	HER2 IHC	HER2 IHC P (3+/ 2+); N (0/ 1+)	HER2 FISH	HER2 NGS	Node Metastasis	One-Year Survival
1	Mixed	NM	14	papillary	2	0	N	Non-A	-	-	D
2	Mixed	NM	14	papillary	2	0	N	Non-A	-	N	D
3	Mixed	NM	12	papillary	2	2+	P	Amp	-	N	A
4	Mixed	NM	6	papillary	2	0	N	Non-A	WT	P	D
5	Bull terrier	SF	10	papillary	2	0	N	Non-A	WT	-	-
6	Mixed	SF	12	papillary	2	2+	P	Non-A	-	N	D
7	Mixed	SF	13	squamous	3	1+	N	Non-A	WT	P	A
8	Weimaraner	NM	10	papillary	2	2+	P	Non-A	WT	N	D
9	Bloodhound	SF	16	papillary	1	2+	P	Non-A	-	-	D
10	Fox terrier	F	13	papillary	1	3+	P	Amp	V659E (VAF 29%)	-	-
11	Mixed	SF	11	squamous	2	3+	P	Non-A	WT	-	D
12	Rottweiler	F	9	micropapillary	2	2+	P	Non-A	WT	N	D
13	Fox Terrier	M	12	papillary	1	1+	N	Non-A	-	-	A
14	Labrador Retriever	M	11	papillary	1	1+	N	Non-A	WT	N	A
15	Labrador Retriever	SF	11	lepidic	1	0	N	-	WT	N	D
16	Mixed	NM	15	lepidic	1	1+	N	Non-A	WT	-	D
17	Shih-Tzu	NM	11	squamous	2	2+	P	-	-	-	D
18	Chihuahua	NM	10	papillary	1	0	N	Non-A	WT	N	D
19	Mixed	F	10	papillary	3	3+	P	-	WT	N	D

Abbreviations: M = male; F = female; NM = neutered male; SF = spayed female; Pos = positive; Neg = negative; Amp = amplified; Non-A = non-amplified; - = non-evaluable; WT = wild type; D = dead; A = alive.

## Data Availability

The original contributions presented in the study are included in the article, further inquiries can be directed to the corresponding author.
